# Construction of the membership surface of imprecise vector

**DOI:** 10.1186/2193-1801-3-722

**Published:** 2014-12-10

**Authors:** Dhruba Das, Hemanta K Baruah

**Affiliations:** Department of Statistics, Gauhati University, Guwahati, 781014 Assam India; Vice chancellor, Bodoland University, Kokrajhar, 783370 Assam India

**Keywords:** Membership function, Imprecise vector, Dubois-Prade left and right reference functions, Distribution function, Density function

## Abstract

In this article, a method has been developed to construct the membership surface of imprecise vector based on Randomness-Impreciseness Consistency Principle. The Randomness-Impreciseness Consistency Principle leads to define a normal law of impreciseness using two different laws of randomness. The Dubois-Prade left and right reference functions of an imprecise number are *distribution function* and complementary *distribution function* respectively. In this article, based on the Randomness-Impreciseness Consistency Principle we have successfully obtained the membership surface of imprecise vector and demonstrated with the help of numerical examples.

## 1. Introduction

Dubois and Prade (Kaufmann and Gupta 1984) have defined a fuzzy number *X* = [*a*, *b*, *c*] with membership function


*L*(*x*) being a continuous non-decreasing function in the interval [a, b], and *R*(*x*) being a continuous non-increasing function in the interval [b, c], with *L*(*a*) = *R*(*c*) = 0 and *L*(*b*) = *R*(*b*) = 1. Dubois and Prade named *L*(*x*) as left reference function and *R*(*x*) as right reference function of the concerned fuzzy number. A continuous non-decreasing function of this type is also called a distribution function with reference to a Lebesgue-Stieltjes measure (De Barra 1987).

In this article, on the simple assumption that the Dubois-Prade left reference function is a distribution function, and similarly the Dubois-Prade right reference function is a complementary distribution function, we are going to demonstrate the method of obtaining the membership surface of an imprecise vector. Here the term imprecise is used instead of fuzzy because, in the Zadehian theory of fuzzy sets there are two flaws (Baruah 2011a, Baruah 2012). First, it had been accepted that the fuzzy sets do not in any way conform to the classical measure theoretic formalisms. Secondly, it had been agreed upon that given a fuzzy set neither its intersection with its complement is the null set, nor its union with the complement is the universal set. The Zadehian definition of complement of a fuzzy set is defective (Baruah 1999). In the Zadehian definition of complementation, fuzzy membership function and fuzzy membership value have been taken to be the same, and that is where the defect lies. Indeed fuzzy membership function and fuzzy membership value are two different things for the complement of a normal fuzzy set (Baruah 2011a). Instead of saying (Baruah 2011b; Baruah 2011c) that the mathematics of fuzziness has been incorrectly explained, Baruah has started the whole process anew, introducing the *theory of imprecise sets*, which might initially look similar to the theory of fuzzy sets.

## 2. The mathematical explanation of imprecise vector

**Figure 1:**
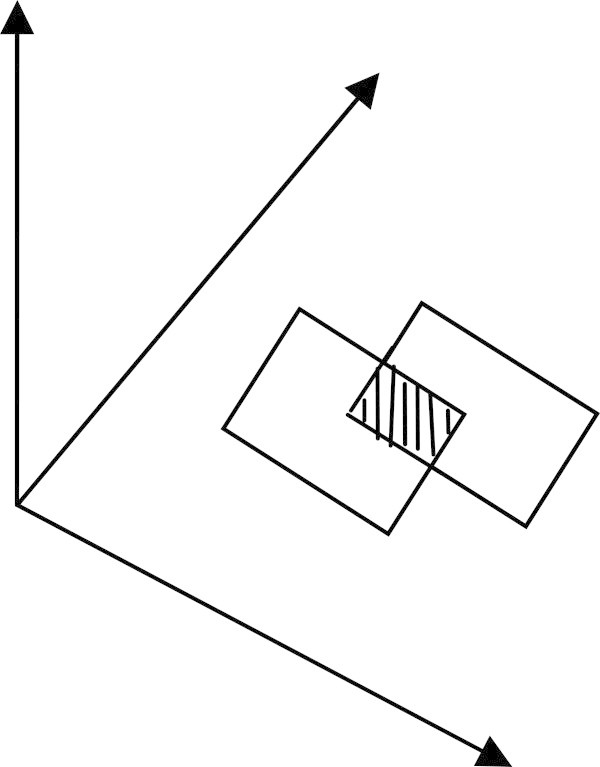
**Superimposition of two plates.**

Baruah (2011b) has successfully shown the construction of a normal imprecise number with the help of the operation of superimposition of real intervals. Das et al. (2013) has shown the construction of normal imprecise number using data from earthquake waveform and has studied the pattern of the membership curve of the waveform. In this article, instead of superimposing real intervals, it will be discussed about how to obtain the membership surface of a two dimensional imprecise vector if we superimpose some plates in the two dimensional plane. In Figure 1 two plates are superimposed restricting the condition that the intersection of the two plates is not void.

We can easily visualize in Figure 1, that the probability of the shaded area is 1 and the probability for the unshaded area of the plates will be ½. But if the number of superimposition is large then it will be very difficult to obtain the probabilities by simply observing the imposition of the plates. So, in that situation a different technique can be used to obtain the probabilities when the number of operation of superimposition is very large. At first, it is discussed, about the operation of superimposition in the two dimensional case when the variable *X* is imprecise but *Y* is not imprecise.

### The operation of superimposition

The operation of superimposition of two real intervals [(*a*_1_, 0), (*b*_1_, 0)] and [(*a*_2_, 0), (*b*_2_, 0)] as


where (*a*_(1)_, 0) = min[(*a*_1_, 0), (*a*_2_, 0)] , (*a*_(2)_, 0) = max[(*a*_1_, 0), (*a*_2_, 0)] , (*b*_(1)_, 0) = min[(*b*_1_, 0), (*b*_2_, 0)] and (*b*_(2)_, 0) = max[(*b*_1_, 0), (*b*_2_, 0)]. Here we have assumed without any loss of generality that [(*a*_1_, 0), (*b*_1_, 0)] ∩ [(*a*_2_, 0), (*b*_2_, 0)] is not void or in other words that max[(*a*_*i*_, 0)] ≤ min[(*b*_*i*_, 0)], *i* = 1, 2.

**Figure 2:**
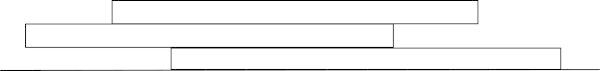
**Superimposition of**  **and**  **.**

**Figure 3:**
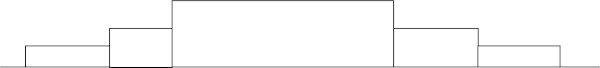
**Cumulative and complementary cumulative distribution functions.**

**Figure 4:**
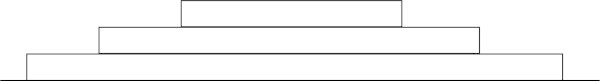
**Discrete Dubois- Prade left and right reference functions.**



For the three intervals  and  all with elements with a constant level of partial presence equal to 1/3 everywhere (See Figures 2, 3 and 4), we shall have

where, for example  represents the interval [(*y*_(1)_, 0), (*y*_(2)_, 0)] with level of partial presence 2/3 for all elements in the entire interval, (*x*_(1)_, 0), (*x*_(2)_, 0), (*x*_(3)_, 0) be the values of (*x*_1_, 0), (*x*_2_, 0), (*x*_3_, 0) arranged in increasing order of magnitude, and similarly (*y*_(1)_, 0), (*y*_(2)_, 0), (*y*_(3)_, 0) be the values of (*y*_1_, 0), (*y*_2_, 0), (*y*_3_, 0) arranged in increasing order of magnitude again. We here presumed that [(*x*_1_, 0), (*y*_1_, 0)] ∩ [(*x*_2_, 0), (*y*_2_, 0)] ∩ [(*x*_3_, 0), (*y*_3_, 0)] is not void. It can be seen that for n imprecise intervals , all with membership value are equal to  everywhere, we shall have


where for example  represents the uniformly imprecise interval [(*b*_(1)_, 0), (*b*_(2)_, 0)] with membership  in the entire interval, (*a*_(1)_, 0), (*a*_(2)_, 0), …, (*a*_(*n*)_, 0) be the values of (*a*_1_, 0), (*a*_2_, 0), …, (*a*_*n*_, 0) arranged in increasing order of magnitude and (*b*_(1)_, 0), (*b*_(2)_, 0), …, (*b*_(*n*)_, 0) be the values of (*b*_1_, 0), (*b*_2_, 0), …, (*b*_*n*_, 0) arranged in increasing order of magnitude. Thus for the imprecise intervals  all with uniform membership , the values of membership of the superimposed imprecise intervals are  and . These values of membership considered in two halves as


and


would suggest that they can define an empirical distribution and a complementary empirical distribution on (*x*_11_, 0), (*x*_12_, 0), …, (*x*_1*n*_, 0) and (*x*_21_, 0), (*x*_22_, 0), …, (*x*_2*n*_, 0) respectively. In other words, for realizations of the values of (*x*_(11)_, 0), (*x*_(12)_, 0), …, (*x*_(1*n*)_, 0) are in increasing order and of (*x*_(21)_, 0), (*x*_(22)_, 0), …, (*x*_(2*n*)_, 0) again are in increasing order, we can see that if we define


then the Glivenko – Cantelli Lemma on Order Statistics assures that


where ∏_1_[(*a*, 0), (*x*_1_, 0)], (*a*, 0) ≤ (*x*_1_, 0) ≤ (*b*, 0) and *ψ*_2_(*x*_*2*_, 0), (*b*, 0) ≤ (*x*_*2*_, 0) ≤ (*c*, 0) *ψ*_2_(*x*_2_, 0), (*b*, 0) ≤ (*x*_2_, 0) ≤ (*c*, 0) are two probability distributions. Thus


where


Thus, if *φ*_1_(*x*, 0) and (1 - *φ*_2_(*x*, 0)) are two independent probability distribution functions defined in [(*α*, 0), (*β*, 0)] and [(*β*, 0), (*γ*, 0)] respectively, then the membership surface of a normal imprecise vector *N* = [(*α*, 0), (*β*, 0), (*γ*, 0)] can be expressed as


or


Here, in the case of two dimensions we have considered that the value of *y* is zero. But instead of zero if we consider any precise value of *y,* then in the above membership surface only the value of *y* will be changed.

Similarly, we can also show that, the membership surface of a normal imprecise vector *N* = [(0, *α*), (0, *β*), (0, *γ*)] can be expressed as


Here, we have also considered the value of *x* is zero. But instead of zero if we consider any precise value of *x,* then in the above membership surface only the value of *x* will be changed.

Now, we are going to discuss about the method how to obtain the membership surface of the vector (*X*, *Y*), where the variables *x* and *y* both are imprecise.

Consider an imprecise vector (*X*, *Y*), where *X* and *Y* are imprecise represented by *X* = [*a, b, c*] and *Y* = [*p, q, r*] respectively. Assume that *X* and *Y* are independently distributed. Let the membership function of *X* and *Y* be *μ*_*X*|*Y*_(*x*, *y*) and *μ*_*Y*|*X*_(*x*, *y*) as mentioned below


and


Then the membership surface of the imprecise vector (*X*, *Y*) can be obtained as follows


For a three dimensional imprecise vector (*X*, *Y*, *Z*), where the membership functions of *X*, *Y* and *Z* are as mentioned below:


Then the membership surface of the imprecise vector (*X*, *Y*, *Z*) will be as shown below:


## 3. Numerical Examples

**Example 1.** Let (*X*, *Y*) be an imprecise vector, where both *X* and *Y* are imprecise with imprecise membership functions


and


According to Randomness- Impreciseness Consistency Principle the left reference functions *L*(*x*) = *x* - 1; 1 ≤ *x* ≤ 2, 3 ≤ *y* ≤ 6 and , are distribution functions and the right reference functions  and *R*(*y*) = 6 - *y*; 5 ≤ *y* ≤ 6, 1 ≤ *x* ≤ 4 are complementary distribution functions.

Now, according to our standpoint the membership surface *μ*_*X*,*Y*_(*x*, *y*) of the imprecise vector (*X*, *Y*) can be obtained as follows


**Figure 5:**
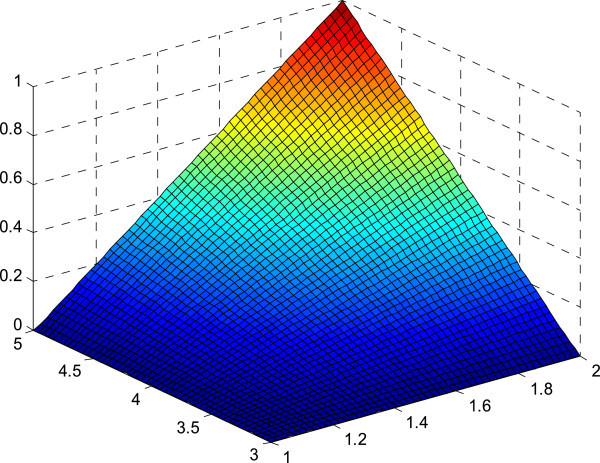
**Membership surface of** ***L(x)L(y)*** **.**

**Figure 6:**
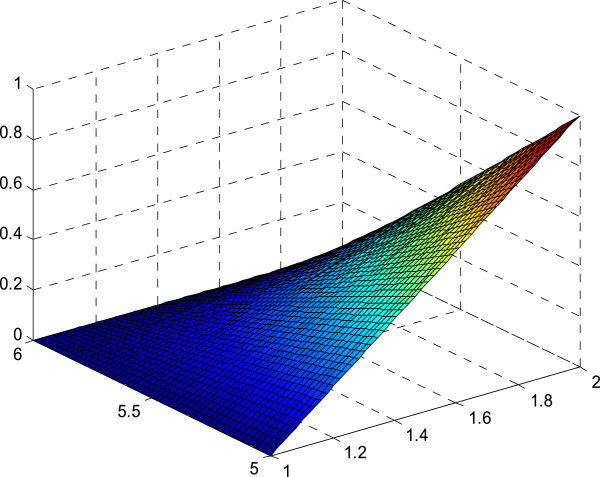
**Membership surface of** ***L(x)R(y)*** **.**

**Figure 7:**
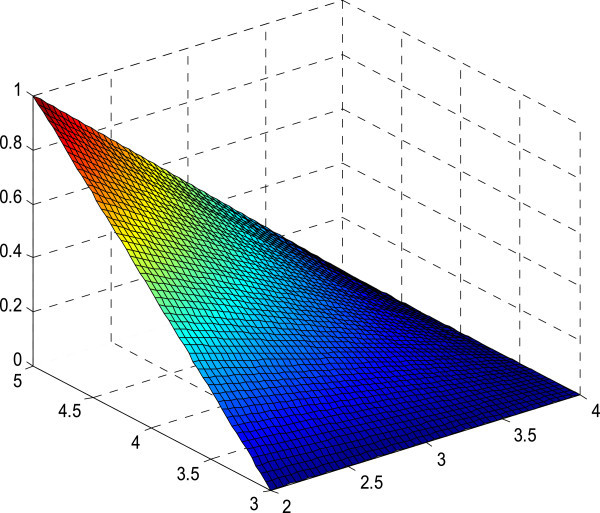
**Membership surface of** ***R(x)L(y)*** **.**

**Figure 8:**
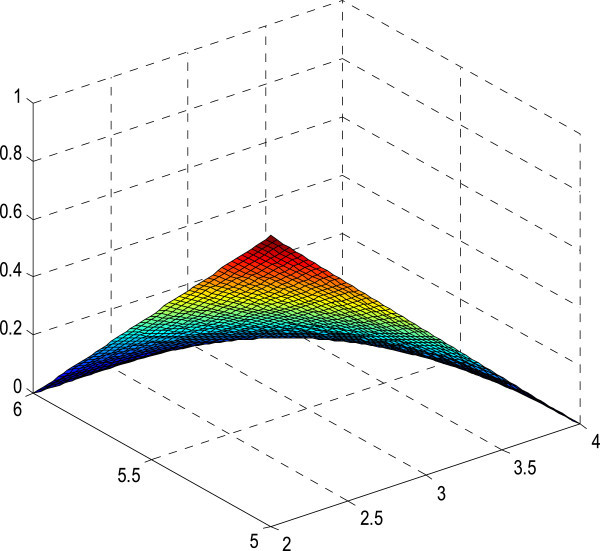
**Membership surface of** ***R(x)R(y)*** **.**

**Figure 9:**
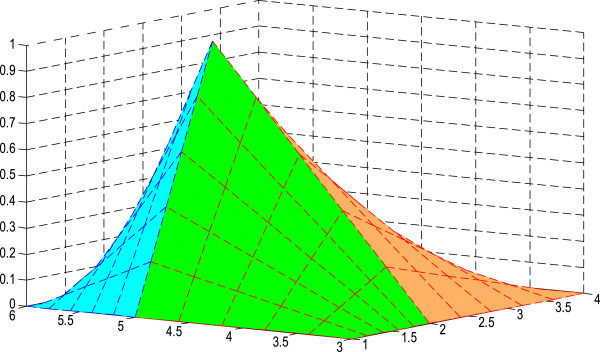
**Membership surface of** ***μ***_***X,Y***_ **(** ***x,*** ***y*** **)** **.**

The figures of the membership surfaces of *L(x)L(y), L(x)R(y), R(x)L(y) and R(x)R(y)* are given in Figures 5, 6, 7 and 8 respectively. The membership surface of the imprecise vector (*X*, *Y*) is shown in Figure 9.

Now, to get the surface section of the membership surface if we cut the membership surface of the imprecise vector, which is in the two dimensions is nothing but the membership function of a subnormal imprecise number. If we cut the membership surface through the point on which the presence level is one, which is in the two dimensions is nothing but the membership function of a normal imprecise number.

**Example 2.** Let (*X*, *Y, Z*) be an imprecise vector, where *X*, *Y* and *Z* are imprecise with imprecise membership functions


and


Now the membership surface *μ*_*X*,*Y*,*Z*_(*x*, *y*, *z*) of the imprecise vector (*X*, *Y, Z*) can be obtained as follows


## 4. Conclusion

In this article, the method has been shown successfully how to obtain the membership surface of the imprecise vector based on the Randomness- Impreciseness Consistency Principle. Here nothing has been done heuristically. The theory has been successfully developed and demonstrated with the help of numerical examples. Here the method of construction of the membership surface has been studied only for two and three dimensional vectors, but with the help of this method one can easily obtain the membership surface of n-dimensional vector too.
